# Bis(μ-1,2-bis­{[2-(2-pyrid­yl)-1*H-*imidazol-1-yl]meth­yl}benzene)bis­[bis­(thio­cyanato-κ*N*)cadmium(II)]

**DOI:** 10.1107/S1600536810008135

**Published:** 2010-03-06

**Authors:** Hongsheng Liu, Lianjiang Su, Limin Wang, Weihong Li

**Affiliations:** a School of Chemistry and Chemical Engineering, Daqing Normal University, Daqing 163712, People’s Republic of China

## Abstract

The asymmetric unit of the binuclear title compound, [Cd_2_(NCS)_4_(C_24_H_20_N_6_)_2_], contains one half-mol­ecule, consisting of one Cd^2+^ cation, two half 1,2-bis­{[2-(2-pyrid­yl)-1*H*-imidazol-1-yl]meth­yl}benzene (*L*) ligands and two SCN^−^ anions. The dimeric cyclic mol­ecule is completed by crystallographic inversion symmetry. The Cd^2+^ cation is coordinated by two N atoms from two SCN^−^ anions and four N atoms from two symmetry-related *L* ligands, exhibiting a distorted octrahedral coordination. A two-dimensional supra­molecular network stacked parallel to [210] is finally formed by linking these dimers through weak π–π stacking inter­actions between the pyridine rings and benzene rings of adjacent dimers, with a plane-to-plane distance of 3.36 (6) Å and a centroid–centroid distance of 3.67 (2) Å. One of the thio­cyanate S atoms is equally disordered over two positions.

## Related literature

For general background to the luminescent properties of cadmium compounds, see: Yam & Lo (1999 ▶); Zheng *et al.* (2004 ▶). For related structures, see: Dai *et al.* (2002 ▶); Luan *et al.* (2006 ▶); Wang *et al.* (2003 ▶).
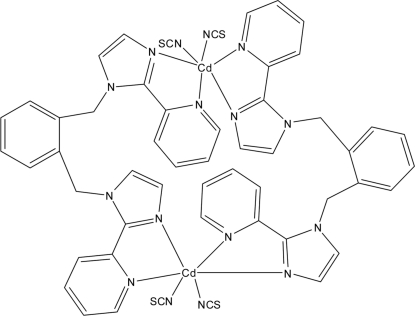


## Experimental

### 

#### Crystal data

[Cd_2_(NCS)_4_(C_24_H_20_N_6_)_2_]
*M* *_r_* = 1242.04
Monoclinic, 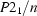

*a* = 10.1170 (5) Å
*b* = 24.0740 (12) Å
*c* = 10.723 (1) Å
β = 97.678 (1)°
*V* = 2588.2 (3) Å^3^
*Z* = 2
Mo *K*α radiation
μ = 1.04 mm^−1^
*T* = 293 K
0.33 × 0.31 × 0.28 mm

#### Data collection

Bruker APEX CCD area-detector diffractometer
Absorption correction: multi-scan (*SADABS*; Sheldrick, 1996 ▶) *T*_min_ = 0.717, *T*_max_ = 0.748
15880 measured reflections
6112 independent reflections
2967 reflections with *I* > 2σ(*I*)
*R*_int_ = 0.050

#### Refinement

*R*[*F* ^2^ > 2σ(*F* ^2^)] = 0.040
*wR*(*F* ^2^) = 0.079
*S* = 0.96
6112 reflections
333 parameters
H-atom parameters constrained
Δρ_max_ = 0.70 e Å^−3^
Δρ_min_ = −0.56 e Å^−3^

### 

Data collection: *SMART* (Bruker, 1997 ▶); cell refinement: *SAINT* (Bruker, 1999 ▶); data reduction: *SAINT*; program(s) used to solve structure: *SHELXS97* (Sheldrick, 2008 ▶); program(s) used to refine structure: *SHELXL97* (Sheldrick, 2008 ▶); molecular graphics: *DIAMOND* (Brandenburg & Putz, 2008 ▶); software used to prepare material for publication: *SHELXL97*.

## Supplementary Material

Crystal structure: contains datablocks global, I. DOI: 10.1107/S1600536810008135/wm2310sup1.cif

Structure factors: contains datablocks I. DOI: 10.1107/S1600536810008135/wm2310Isup2.hkl

Additional supplementary materials:  crystallographic information; 3D view; checkCIF report

## Figures and Tables

**Table 1:** Selected bond lengths (Å)

N1—Cd1	2.523 (3)
N2—Cd1	2.289 (3)
N5—Cd1	2.313 (3)
N6—Cd1	2.420 (3)
N7—Cd1	2.238 (4)
N8—Cd1	2.291 (4)

**Fig. 1.**
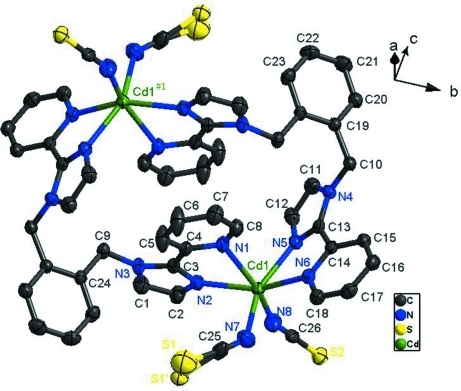
A displacement ellipsoids view of (I), drawn at 30% probability level, showing two cations and one anion. All non-labelled atoms are generated by symmetry operator: 2-x, y, 1-z. H atoms were omitted for clarity. The two orientations of the disordered thiocyanate anion are shown.

**Fig. 2.**
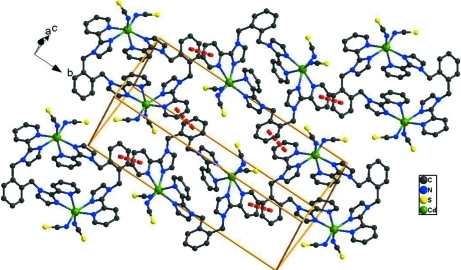
View of the two-dimensional supramolecular structure formed by π–π stacking interactions (red dashed lines).

## supplementary crystallographic information

### Comment

Interest in cadmium compounds was provoked by their luminescent properties (Yam & Lo, 1999; Zheng *et al.*, 2004). A number of cadmium compounds have been reported with different N-donor ligands. In this paper, we present the hydrothermal synthesis and crystal structure of the title compound, (I), [Cd_2_(C_24_H_20_N_6_)_2_(SCN)_4_], based on the 1,2-bis{[2-(2-pyridyl)-1*H-*imidazol-1-yl]methyl}benzene ligand (hereafter *L*).

The asymmetric unit of (I) contains one Cd^2+^ cation, two halfs of the *L* ligand and two SCN^-^ anions. Two complete *L* ligands link two Cd^2+^ cations to form a centrosymmetric dimeric ring. So the asymmetric unit contains only half of the ring molecule (Fig. 1). The Cd^2+^ cation is coordinated to the N atom of two SCN^-^ anions and four N atoms from symmetry-related *L* ligands within normal Cd—N distances (Dai *et al.*, 2002; Luan *et al.*, 2006; Wang *et al.*, 2003). The resulting CdN_6_ polyhedron can be considered as a distorted octahedron. Each dimer links adjacent dimers *via*π–π interactions between pyridine rings and benzene rings to form a 2D supramolecular network stacked along [210] (Fig. 2), with a plane to plane distance of 3.36 (6) Å and a centroid-centroid distance of 3.67 (2) Å.

### Experimental

A mixture of Cd(OAc)_2_^.^2H_2_O (0.13 g, 0.50 mmol), *L* (0.2 g, 0.5 mmol), KSCN (0.10 g, 1 mmol) and H_2_O (10 ml) was stirred for 1 h, and then transferred and sealed in a 25 ml Teflon-lined stainless steel container. The container was heated to 423 K, held at that temperature for 72 h, and cooled to room temperature at a rate of 10 Kh^-1^. Colourless parallelepipeds of (I) were collected in 78% yield.

### Refinement

One of the SCN^-^ groups is disordered over two positions. The S atom was refined with a 0.5:0.5 occupancy ratio. All H atoms on C atoms were positioned geometrically and refined as riding atoms, with C—H = 0.93 Å for aromatic C atoms, and with C—H = 0.97 Å for aliphatic C atoms, and *U*_iso_=1.2 or 1.5*U*_eq_(C).

### Crystal data

**Table:**

[Cd_2_(NCS)_4_(C_24_H_20_N_6_)_2_]	*F*(000) = 1248
*M**_r_* = 1242.04	*D*_x_ = 1.594 Mg m^−^^3^
Monoclinic, *P*2_1_/*n*	Mo *K*α radiation, λ = 0.71069 Å
Hall symbol: -P 2yn	Cell parameters from 159 reflections
*a* = 10.1170 (5) Å	θ = 1.7–28.3°
*b* = 24.0740 (12) Å	µ = 1.04 mm^−^^1^
*c* = 10.723 (1) Å	*T* = 293 K
β = 97.678 (1)°	Block, colorless
*V* = 2588.2 (3) Å^3^	0.33 × 0.31 × 0.28 mm
*Z* = 2	

### Data collection

**Table:**

Bruker APEX CCD area-detector diffractometer	6112 independent reflections
Radiation source: fine-focus sealed tube	2967 reflections with *I* > 2σ(*I*)
graphite	*R*_int_ = 0.050
ω scans	θ_max_ = 28.3°, θ_min_ = 1.7°
Absorption correction: multi-scan (*SADABS*; Sheldrick, 1996)	*h* = −13→13
*T*_min_ = 0.717, *T*_max_ = 0.748	*k* = −31→16
15880 measured reflections	*l* = −11→13

### Refinement

**Table:**

Refinement on *F*^2^	Primary atom site location: structure-invariant direct methods
Least-squares matrix: full	Secondary atom site location: difference Fourier map
*R*[*F*^2^ > 2σ(*F*^2^)] = 0.040	Hydrogen site location: inferred from neighbouring sites
*wR*(*F*^2^) = 0.079	H-atom parameters constrained
*S* = 0.96	*w* = 1/[σ^2^(*F*_o_^2^) + (0.0255*P*)^2^] where *P* = (*F*_o_^2^ + 2*F*_c_^2^)/3
6112 reflections	(Δ/σ)_max_ = 0.001
333 parameters	Δρ_max_ = 0.70 e Å^−^^3^
0 restraints	Δρ_min_ = −0.56 e Å^−^^3^

### Special details

**Table:**

Geometry. All esds (except the esd in the dihedral angle between two l.s. planes) are estimated using the full covariance matrix. The cell esds are taken into account individually in the estimation of esds in distances, angles and torsion angles; correlations between esds in cell parameters are only used when they are defined by crystal symmetry. An approximate (isotropic) treatment of cell esds is used for estimating esds involving l.s. planes.
Refinement. Refinement of F^2^ against ALL reflections. The weighted R-factor wR and goodness of fit S are based on F^2^, conventional R-factors R are based on F, with F set to zero for negative F^2^. The threshold expression of F^2^ > 2sigma(F^2^) is used only for calculating R-factors(gt) etc. and is not relevant to the choice of reflections for refinement. R-factors based on F^2^ are statistically about twice as large as those based on F, and R- factors based on ALL data will be even larger.

### Fractional atomic coordinates and isotropic or equivalent isotropic displacement parameters (Å^2^)

**Table:**

	*x*	*y*	*z*	*U*_iso_*/*U*_eq_	Occ. (<1)
C1	0.6018 (4)	−0.05645 (17)	0.3451 (4)	0.0634 (11)	
H1	0.5438	−0.0863	0.3463	0.076*	
C2	0.5726 (4)	−0.00252 (17)	0.3591 (4)	0.0625 (11)	
H2	0.4892	0.0111	0.3710	0.075*	
C3	0.7782 (4)	−0.00550 (15)	0.3340 (3)	0.0419 (9)	
C4	0.9121 (4)	0.01421 (15)	0.3240 (3)	0.0474 (9)	
C5	1.0101 (4)	−0.01509 (18)	0.2744 (5)	0.0873 (15)	
H5	0.9918	−0.0501	0.2396	0.105*	
C6	1.1351 (5)	0.00779 (19)	0.2766 (5)	0.1046 (18)	
H6	1.2023	−0.0121	0.2455	0.126*	
C7	1.1597 (4)	0.05930 (18)	0.3240 (4)	0.0701 (12)	
H7	1.2445	0.0749	0.3296	0.084*	
C8	1.0561 (4)	0.08777 (16)	0.3635 (4)	0.0607 (11)	
H8	1.0711	0.1242	0.3907	0.073*	
C9	0.8023 (3)	−0.11083 (13)	0.3109 (3)	0.0483 (10)	
H9A	0.7642	−0.1399	0.3577	0.058*	
H9B	0.8952	−0.1068	0.3462	0.058*	
C10	1.0699 (3)	0.21467 (13)	0.7506 (3)	0.0465 (9)	
H10A	1.0528	0.2507	0.7855	0.056*	
H10B	1.1256	0.2202	0.6847	0.056*	
C11	0.8661 (4)	0.15565 (15)	0.7598 (4)	0.0536 (10)	
H11	0.8791	0.1483	0.8457	0.064*	
C12	0.7688 (3)	0.13491 (15)	0.6753 (4)	0.0550 (11)	
H12	0.7029	0.1104	0.6935	0.066*	
C13	0.8852 (3)	0.18861 (14)	0.5747 (3)	0.0432 (9)	
C14	0.9230 (3)	0.22131 (14)	0.4689 (3)	0.0440 (9)	
C15	0.9941 (3)	0.27037 (15)	0.4804 (4)	0.0515 (10)	
H15	1.0227	0.2852	0.5594	0.062*	
C16	1.0223 (3)	0.29724 (15)	0.3729 (4)	0.0575 (11)	
H16	1.0713	0.3300	0.3786	0.069*	
C17	0.9765 (4)	0.27452 (17)	0.2572 (4)	0.0621 (11)	
H17	0.9960	0.2912	0.1836	0.075*	
C18	0.9010 (4)	0.22634 (16)	0.2524 (4)	0.0558 (10)	
H18	0.8680	0.2118	0.1741	0.067*	
C19	1.1433 (3)	0.17880 (14)	0.8525 (3)	0.0432 (9)	
C20	1.1505 (4)	0.19548 (16)	0.9760 (4)	0.0568 (10)	
H20	1.1086	0.2283	0.9946	0.068*	
C21	1.2183 (4)	0.16470 (18)	1.0729 (4)	0.0679 (12)	
H21	1.2223	0.1767	1.1558	0.082*	
C22	1.2797 (4)	0.11638 (18)	1.0455 (4)	0.0704 (12)	
H22	1.3261	0.0955	1.1101	0.084*	
C23	1.2730 (4)	0.09839 (15)	0.9220 (4)	0.0584 (11)	
H23	1.3150	0.0655	0.9040	0.070*	
C24	0.7961 (3)	−0.12925 (14)	0.1749 (3)	0.0418 (9)	
C25	0.6902 (5)	0.09909 (19)	0.0716 (4)	0.0749 (14)	
C26	0.4193 (4)	0.16946 (16)	0.4024 (4)	0.0528 (10)	
N1	0.9342 (3)	0.06631 (12)	0.3652 (3)	0.0532 (8)	
N2	0.6815 (3)	0.02887 (12)	0.3535 (3)	0.0487 (8)	
N3	0.7331 (3)	−0.05881 (12)	0.3288 (2)	0.0465 (8)	
N4	0.9427 (3)	0.18963 (12)	0.6960 (3)	0.0438 (7)	
N5	0.7813 (3)	0.15517 (12)	0.5587 (3)	0.0494 (8)	
N6	0.8737 (3)	0.20017 (12)	0.3549 (3)	0.0505 (8)	
N7	0.6949 (4)	0.12982 (16)	0.1462 (4)	0.0913 (13)	
N8	0.5058 (3)	0.14232 (15)	0.3802 (4)	0.0835 (12)	
S1	0.7164 (4)	0.05643 (17)	−0.0369 (4)	0.1451 (15)*	0.50
S1'	0.6395 (3)	0.05156 (10)	−0.0503 (2)	0.0658 (7)*	0.50
S2	0.29671 (10)	0.20853 (4)	0.43058 (10)	0.0628 (3)	
Cd1	0.72240 (3)	0.122387 (11)	0.35612 (3)	0.05177 (11)	

### Atomic displacement parameters (Å^2^)

**Table:**

	*U*^11^	*U*^22^	*U*^33^	*U*^12^	*U*^13^	*U*^23^
C1	0.050 (3)	0.059 (3)	0.083 (3)	−0.011 (2)	0.017 (2)	−0.001 (2)
C2	0.046 (3)	0.055 (3)	0.088 (3)	0.007 (2)	0.013 (2)	−0.010 (2)
C3	0.046 (2)	0.036 (2)	0.045 (2)	0.0001 (18)	0.0092 (18)	−0.0034 (17)
C4	0.057 (3)	0.036 (2)	0.050 (2)	0.0035 (19)	0.0096 (19)	−0.0005 (19)
C5	0.069 (3)	0.053 (3)	0.150 (4)	−0.010 (2)	0.054 (3)	−0.029 (3)
C6	0.076 (4)	0.058 (3)	0.193 (6)	−0.009 (3)	0.065 (4)	−0.036 (3)
C7	0.052 (3)	0.058 (3)	0.104 (3)	−0.002 (2)	0.025 (2)	0.003 (3)
C8	0.057 (3)	0.043 (3)	0.081 (3)	−0.006 (2)	0.008 (2)	−0.005 (2)
C9	0.056 (2)	0.035 (2)	0.054 (2)	−0.0002 (17)	0.0100 (19)	0.0008 (18)
C10	0.048 (2)	0.039 (2)	0.051 (2)	−0.0066 (17)	0.0033 (18)	−0.0084 (18)
C11	0.050 (2)	0.057 (3)	0.056 (3)	0.000 (2)	0.012 (2)	−0.003 (2)
C12	0.044 (2)	0.054 (3)	0.070 (3)	−0.0087 (19)	0.017 (2)	−0.007 (2)
C13	0.047 (2)	0.033 (2)	0.049 (2)	0.0052 (17)	0.0024 (19)	−0.0047 (18)
C14	0.040 (2)	0.034 (2)	0.056 (3)	0.0045 (16)	0.0004 (18)	−0.0026 (19)
C15	0.050 (2)	0.040 (2)	0.062 (3)	−0.0024 (18)	−0.006 (2)	−0.002 (2)
C16	0.054 (3)	0.042 (3)	0.074 (3)	−0.0046 (18)	−0.003 (2)	0.007 (2)
C17	0.067 (3)	0.058 (3)	0.061 (3)	0.006 (2)	0.006 (2)	0.012 (2)
C18	0.060 (3)	0.047 (3)	0.058 (3)	0.004 (2)	0.000 (2)	−0.001 (2)
C19	0.045 (2)	0.040 (2)	0.044 (2)	−0.0025 (17)	0.0036 (17)	−0.0057 (19)
C20	0.057 (3)	0.052 (3)	0.060 (3)	−0.002 (2)	0.004 (2)	−0.012 (2)
C21	0.079 (3)	0.080 (4)	0.046 (3)	−0.005 (3)	0.010 (2)	−0.004 (2)
C22	0.076 (3)	0.080 (4)	0.052 (3)	0.004 (3)	−0.001 (2)	0.011 (3)
C23	0.068 (3)	0.046 (2)	0.061 (3)	0.003 (2)	0.009 (2)	0.012 (2)
C24	0.044 (2)	0.036 (2)	0.046 (2)	−0.0075 (17)	0.0049 (17)	0.0002 (18)
C25	0.110 (4)	0.062 (3)	0.055 (3)	0.020 (3)	0.020 (3)	0.014 (2)
C26	0.047 (3)	0.044 (3)	0.063 (3)	−0.0120 (19)	−0.012 (2)	0.005 (2)
N1	0.052 (2)	0.040 (2)	0.068 (2)	−0.0014 (15)	0.0071 (16)	−0.0059 (16)
N2	0.0443 (19)	0.0429 (19)	0.059 (2)	−0.0029 (15)	0.0063 (16)	−0.0059 (16)
N3	0.049 (2)	0.0359 (19)	0.057 (2)	0.0000 (15)	0.0143 (16)	−0.0063 (15)
N4	0.0421 (18)	0.0418 (19)	0.0469 (19)	−0.0036 (14)	0.0034 (15)	−0.0023 (15)
N5	0.0431 (19)	0.043 (2)	0.061 (2)	−0.0036 (15)	0.0018 (16)	−0.0008 (16)
N6	0.054 (2)	0.0395 (19)	0.057 (2)	0.0040 (15)	0.0019 (17)	−0.0005 (17)
N7	0.119 (3)	0.083 (3)	0.066 (3)	−0.013 (3)	−0.008 (2)	−0.004 (2)
N8	0.051 (2)	0.071 (3)	0.125 (3)	0.0068 (19)	−0.003 (2)	−0.018 (2)
S2	0.0602 (7)	0.0624 (8)	0.0662 (7)	0.0067 (5)	0.0099 (6)	−0.0016 (6)
Cd1	0.04836 (17)	0.03862 (17)	0.06495 (19)	0.00251 (14)	−0.00488 (12)	−0.00671 (16)

### Geometric parameters (Å, °)

**Table:**

C1—C2	1.344 (5)	C14—N6	1.357 (4)
C1—N3	1.364 (4)	C14—C15	1.379 (4)
C1—H1	0.9300	C15—C16	1.385 (5)
C2—N2	1.343 (4)	C15—H15	0.9300
C2—H2	0.9300	C16—C17	1.378 (5)
C3—N2	1.319 (4)	C16—H16	0.9300
C3—N3	1.361 (4)	C17—C18	1.386 (5)
C3—C4	1.453 (5)	C17—H17	0.9300
C4—N1	1.339 (4)	C18—N6	1.327 (4)
C4—C5	1.380 (5)	C18—H18	0.9300
C5—C6	1.376 (5)	C19—C20	1.376 (4)
C5—H5	0.9300	C19—C24^i^	1.390 (4)
C6—C7	1.351 (5)	C20—C21	1.382 (5)
C6—H6	0.9300	C20—H20	0.9300
C7—C8	1.366 (5)	C21—C22	1.369 (5)
C7—H7	0.9300	C21—H21	0.9300
C8—N1	1.340 (4)	C22—C23	1.387 (5)
C8—H8	0.9300	C22—H22	0.9300
C9—N3	1.460 (4)	C23—C24^i^	1.388 (5)
C9—C24	1.517 (4)	C23—H23	0.9300
C9—H9A	0.9700	C24—C23^i^	1.388 (5)
C9—H9B	0.9700	C24—C19^i^	1.390 (4)
C10—N4	1.470 (4)	C25—N7	1.087 (5)
C10—C19	1.508 (4)	C25—S1	1.600 (6)
C10—H10A	0.9700	C25—S1'	1.761 (6)
C10—H10B	0.9700	C26—N8	1.143 (4)
C11—C12	1.342 (4)	C26—S2	1.617 (4)
C11—N4	1.372 (4)	N1—Cd1	2.523 (3)
C11—H11	0.9300	N2—Cd1	2.289 (3)
C12—N5	1.363 (4)	N5—Cd1	2.313 (3)
C12—H12	0.9300	N6—Cd1	2.420 (3)
C13—N5	1.317 (4)	N7—Cd1	2.238 (4)
C13—N4	1.352 (4)	N8—Cd1	2.291 (4)
C13—C14	1.473 (5)		
			
C2—C1—N3	106.7 (3)	C17—C18—H18	118.6
C2—C1—H1	126.7	C20—C19—C24^i^	119.1 (3)
N3—C1—H1	126.7	C20—C19—C10	119.2 (3)
N2—C2—C1	110.2 (3)	C24^i^—C19—C10	121.8 (3)
N2—C2—H2	124.9	C19—C20—C21	121.6 (4)
C1—C2—H2	124.9	C19—C20—H20	119.2
N2—C3—N3	110.3 (3)	C21—C20—H20	119.2
N2—C3—C4	121.7 (3)	C22—C21—C20	119.2 (4)
N3—C3—C4	128.0 (3)	C22—C21—H21	120.4
N1—C4—C5	120.7 (4)	C20—C21—H21	120.4
N1—C4—C3	113.4 (3)	C21—C22—C23	120.3 (4)
C5—C4—C3	125.9 (4)	C21—C22—H22	119.9
C6—C5—C4	119.6 (4)	C23—C22—H22	119.9
C6—C5—H5	120.2	C22—C23—C24^i^	120.2 (4)
C4—C5—H5	120.2	C22—C23—H23	119.9
C7—C6—C5	119.8 (4)	C24^i^—C23—H23	119.9
C7—C6—H6	120.1	C23^i^—C24—C19^i^	119.6 (3)
C5—C6—H6	120.1	C23^i^—C24—C9	121.0 (3)
C6—C7—C8	117.8 (4)	C19^i^—C24—C9	119.4 (3)
C6—C7—H7	121.1	N7—C25—S1	167.8 (6)
C8—C7—H7	121.1	N7—C25—S1'	165.7 (5)
N1—C8—C7	123.8 (4)	S1—C25—S1'	26.29 (17)
N1—C8—H8	118.1	N8—C26—S2	178.6 (4)
C7—C8—H8	118.1	C4—N1—C8	118.0 (3)
N3—C9—C24	114.7 (3)	C4—N1—Cd1	112.7 (2)
N3—C9—H9A	108.6	C8—N1—Cd1	124.9 (2)
C24—C9—H9A	108.6	C3—N2—C2	106.5 (3)
N3—C9—H9B	108.6	C3—N2—Cd1	118.8 (2)
C24—C9—H9B	108.6	C2—N2—Cd1	134.5 (3)
H9A—C9—H9B	107.6	C3—N3—C1	106.4 (3)
N4—C10—C19	112.0 (3)	C3—N3—C9	130.5 (3)
N4—C10—H10A	109.2	C1—N3—C9	123.0 (3)
C19—C10—H10A	109.2	C13—N4—C11	105.6 (3)
N4—C10—H10B	109.2	C13—N4—C10	129.2 (3)
C19—C10—H10B	109.2	C11—N4—C10	124.7 (3)
H10A—C10—H10B	107.9	C13—N5—C12	105.7 (3)
C12—C11—N4	107.3 (3)	C13—N5—Cd1	115.6 (2)
C12—C11—H11	126.3	C12—N5—Cd1	134.0 (2)
N4—C11—H11	126.3	C18—N6—C14	118.5 (3)
C11—C12—N5	109.7 (3)	C18—N6—Cd1	125.0 (3)
C11—C12—H12	125.2	C14—N6—Cd1	116.2 (2)
N5—C12—H12	125.2	C25—N7—Cd1	132.4 (4)
N5—C13—N4	111.7 (3)	C26—N8—Cd1	156.1 (3)
N5—C13—C14	120.8 (3)	N7—Cd1—N2	93.92 (12)
N4—C13—C14	127.4 (3)	N7—Cd1—N8	95.98 (15)
N6—C14—C15	121.7 (3)	N2—Cd1—N8	91.91 (12)
N6—C14—C13	113.0 (3)	N7—Cd1—N5	154.04 (13)
C15—C14—C13	125.1 (3)	N2—Cd1—N5	111.75 (10)
C14—C15—C16	119.3 (3)	N8—Cd1—N5	87.30 (12)
C14—C15—H15	120.4	N7—Cd1—N6	85.78 (13)
C16—C15—H15	120.4	N2—Cd1—N6	151.01 (10)
C17—C16—C15	118.8 (4)	N8—Cd1—N6	116.98 (11)
C17—C16—H16	120.6	N5—Cd1—N6	69.97 (10)
C15—C16—H16	120.6	N7—Cd1—N1	94.16 (13)
C16—C17—C18	118.9 (4)	N2—Cd1—N1	68.00 (10)
C16—C17—H17	120.5	N8—Cd1—N1	158.07 (12)
C18—C17—H17	120.5	N5—Cd1—N1	92.03 (10)
N6—C18—C17	122.7 (4)	N6—Cd1—N1	83.09 (10)
N6—C18—H18	118.6		
			
N3—C1—C2—N2	−0.7 (5)	C11—C12—N5—C13	−0.9 (4)
N2—C3—C4—N1	14.9 (5)	C11—C12—N5—Cd1	152.6 (3)
N3—C3—C4—N1	−163.3 (3)	C17—C18—N6—C14	−0.6 (5)
N2—C3—C4—C5	−163.2 (4)	C17—C18—N6—Cd1	172.5 (3)
N3—C3—C4—C5	18.5 (6)	C15—C14—N6—C18	3.4 (5)
N1—C4—C5—C6	5.2 (7)	C13—C14—N6—C18	179.7 (3)
C3—C4—C5—C6	−176.8 (4)	C15—C14—N6—Cd1	−170.3 (2)
C4—C5—C6—C7	−1.8 (8)	C13—C14—N6—Cd1	6.0 (4)
C5—C6—C7—C8	−2.8 (8)	S1—C25—N7—Cd1	−94 (2)
C6—C7—C8—N1	4.4 (7)	S1'—C25—N7—Cd1	98.3 (18)
N4—C11—C12—N5	−0.3 (4)	S2—C26—N8—Cd1	77 (17)
N5—C13—C14—N6	−21.0 (5)	C25—N7—Cd1—N2	−14.0 (6)
N4—C13—C14—N6	162.0 (3)	C25—N7—Cd1—N8	−106.4 (6)
N5—C13—C14—C15	155.2 (3)	C25—N7—Cd1—N5	157.5 (5)
N4—C13—C14—C15	−21.8 (5)	C25—N7—Cd1—N6	136.9 (6)
N6—C14—C15—C16	−3.6 (5)	C25—N7—Cd1—N1	54.1 (6)
C13—C14—C15—C16	−179.5 (3)	C3—N2—Cd1—N7	80.3 (3)
C14—C15—C16—C17	1.0 (5)	C2—N2—Cd1—N7	−94.0 (4)
C15—C16—C17—C18	1.6 (6)	C3—N2—Cd1—N8	176.4 (3)
C16—C17—C18—N6	−1.9 (6)	C2—N2—Cd1—N8	2.1 (4)
N4—C10—C19—C20	−108.1 (3)	C3—N2—Cd1—N5	−95.7 (3)
N4—C10—C19—C24^i^	71.8 (4)	C2—N2—Cd1—N5	90.0 (3)
C24^i^—C19—C20—C21	1.2 (5)	C3—N2—Cd1—N6	−8.1 (4)
C10—C19—C20—C21	−178.9 (3)	C2—N2—Cd1—N6	177.6 (3)
C19—C20—C21—C22	−0.2 (6)	C3—N2—Cd1—N1	−12.6 (2)
C20—C21—C22—C23	−0.4 (6)	C2—N2—Cd1—N1	173.1 (4)
C21—C22—C23—C24^i^	−0.1 (6)	C26—N8—Cd1—N7	−100.4 (9)
N3—C9—C24—C23^i^	−1.1 (5)	C26—N8—Cd1—N2	165.5 (9)
N3—C9—C24—C19^i^	−178.8 (3)	C26—N8—Cd1—N5	53.8 (9)
C5—C4—N1—C8	−3.8 (5)	C26—N8—Cd1—N6	−12.1 (10)
C3—C4—N1—C8	178.0 (3)	C26—N8—Cd1—N1	142.5 (8)
C5—C4—N1—Cd1	153.8 (3)	C13—N5—Cd1—N7	−36.8 (4)
C3—C4—N1—Cd1	−24.4 (4)	C12—N5—Cd1—N7	171.7 (3)
C7—C8—N1—C4	−1.1 (6)	C13—N5—Cd1—N2	134.1 (2)
C7—C8—N1—Cd1	−155.7 (3)	C12—N5—Cd1—N2	−17.4 (3)
N3—C3—N2—C2	−0.9 (4)	C13—N5—Cd1—N8	−134.9 (3)
C4—C3—N2—C2	−179.4 (3)	C12—N5—Cd1—N8	73.6 (3)
N3—C3—N2—Cd1	−176.6 (2)	C13—N5—Cd1—N6	−14.9 (2)
C4—C3—N2—Cd1	4.9 (4)	C12—N5—Cd1—N6	−166.4 (3)
C1—C2—N2—C3	0.9 (5)	C13—N5—Cd1—N1	67.0 (2)
C1—C2—N2—Cd1	175.7 (3)	C12—N5—Cd1—N1	−84.5 (3)
N2—C3—N3—C1	0.5 (4)	C18—N6—Cd1—N7	1.3 (3)
C4—C3—N3—C1	178.9 (3)	C14—N6—Cd1—N7	174.6 (3)
N2—C3—N3—C9	−179.1 (3)	C18—N6—Cd1—N2	91.8 (3)
C4—C3—N3—C9	−0.7 (6)	C14—N6—Cd1—N2	−95.0 (3)
C2—C1—N3—C3	0.1 (4)	C18—N6—Cd1—N8	−93.2 (3)
C2—C1—N3—C9	179.7 (3)	C14—N6—Cd1—N8	80.0 (3)
C24—C9—N3—C3	−91.2 (4)	C18—N6—Cd1—N5	−169.2 (3)
C24—C9—N3—C1	89.4 (4)	C14—N6—Cd1—N5	4.0 (2)
N5—C13—N4—C11	−2.0 (4)	C18—N6—Cd1—N1	96.1 (3)
C14—C13—N4—C11	175.2 (3)	C14—N6—Cd1—N1	−90.7 (2)
N5—C13—N4—C10	170.4 (3)	C4—N1—Cd1—N7	−72.4 (3)
C14—C13—N4—C10	−12.5 (6)	C8—N1—Cd1—N7	83.3 (3)
C12—C11—N4—C13	1.3 (4)	C4—N1—Cd1—N2	20.1 (2)
C12—C11—N4—C10	−171.4 (3)	C8—N1—Cd1—N2	175.9 (3)
C19—C10—N4—C13	−140.8 (3)	C4—N1—Cd1—N8	45.0 (4)
C19—C10—N4—C11	30.2 (5)	C8—N1—Cd1—N8	−159.3 (3)
N4—C13—N5—C12	1.8 (4)	C4—N1—Cd1—N5	132.8 (2)
C14—C13—N5—C12	−175.6 (3)	C8—N1—Cd1—N5	−71.4 (3)
N4—C13—N5—Cd1	−157.4 (2)	C4—N1—Cd1—N6	−157.6 (3)
C14—C13—N5—Cd1	25.2 (4)	C8—N1—Cd1—N6	−1.9 (3)

Symmetry codes: (i) −*x*+2, −*y*, −*z*+1.
